# Preoperative pain neuroscience education for shoulder surgery: A case series

**DOI:** 10.4102/sajp.v76i1.1417

**Published:** 2020-08-11

**Authors:** Adriaan Louw, Debra Rico, Leigh Langerwerf, Nicholas Maiers, Ina Diener, Terry Cox

**Affiliations:** 1 Evidence in Motion, San Antonio, United States of America; 2 Department of Physical Therapy, Rockhurst University, Kansas City, United States of America; 3 Butte Premier Physical Therapy, Chico, United States of America; 4 Department of Physical Therapy, Des Moines University, Des Moines, United States of America; 5 Department of Physiotherapy, Stellenbosch University, Cape Town, South Africa; 6 Department of Physical Therapy, Southwest Baptist University, Bolivar, United States of America

**Keywords:** education, surgery, pain, neuroscience, shoulder

## Abstract

**Background:**

Central sensitisation, in addition to high levels of fear-avoidance and pain catastrophisation may exist in a subgroup of patients with shoulder pain. Pain neuroscience education (PNE) has been shown to positively influence sensitivity of the nervous system, as well as reduce fear and catastrophisation prior to lumbar and total knee surgery. To date, no study has examined the application of PNE prior to shoulder surgery.

**Objectives:**

This study examined the response to preoperative PNE in patients preparing for shoulder surgery.

**Method:**

An exploratory pre–post case series was conducted. Twelve patients scheduled for surgery completed various pre-education measurements including shoulder pain, fear-avoidance, pain catastrophisation, beliefs and expectations regarding surgery, active shoulder flexion and pressure pain thresholds for the involved and uninvolved shoulder and the dominant-sided knee. Patients underwent a standard 30-min, one-on-one PNE session with a physiotherapist prior to surgery.

**Results:**

Following education, all measures improved with some failing to reach significance: self-reported pain (*p* = 0.125), pain catastrophisation (*p* = 0.250) and pain pressure threshold of the uninvolved shoulder (*p* = 0.68) and knee (*p* = 0.097). Fear-avoidance (*p* = 0.013), active shoulder flexion (*p* = 0.013) and pain pressure threshold for the involved shoulder (*p* = 0.004) significantly improved.

**Conclusion:**

A small patient group improved beyond minimal detectable change and/or minimal clinical important difference after education. No significant shifts of the preoperative beliefs occurred after education.

**Clinical implications:**

Preoperative PNE may be beneficial to a subgroup of patients scheduled for shoulder surgery.

## Introduction

It is now well established that a patient’s beliefs and cognitions, especially regarding pain, influence his or her pain experience and outcome related to treatment (Kovacs et al. 2011; Vlaeyen & Linton 2000). For example, high levels of fear-avoidance and pain catastrophisation in chronic low back pain have been shown to predict poorer outcomes (Fritz, George & Delitto 2001; Vlaeyen & Linton 2000). In physiotherapy, pain neuroscience education (PNE) is gaining a lot of attention as one way to influence these unhelpful thoughts and beliefs. Pain neuroscience education is an educational approach that uses metaphors, examples and images to explain the biological and physiological processes involved in a pain experience (Moseley 2002). Current best-evidence supports the use of PNE for chronic musculoskeletal pain to decrease pain, disability, fear-avoidance, pain catastrophisation, limited movement and healthcare utilisation (Louw et al. 2016d; Tegner et al. 2018). Furthermore, the efficacy of PNE for chronic musculoskeletal pain increases when used with other therapeutic treatments especially movement, that is, exercise (Louw et al. 2016d; Wood & Hendrick 2019).

Apart from chronic pain, PNE research has also recently shifted to explore its potential benefits in non-chronic pain states. It has been postulated that teaching patients PNE in the acute, subacute, preoperative and even prior to pain experience (healthy individuals) may potentially decrease the chance of developing chronic pain and disability. For example, preoperative PNE for patients undergoing lumbar surgery and total knee replacements in the United States has shown to yield significant improvements in patient surgical experiences and healthcare utilisation at 6-month, 1-year and 3-year follow-up compared with no preoperative PNE (Louw et al. 2014b, 2016a, 2018b, 2019c). Two recent studies explored the application of PNE to acute and subacute low back pain, with one study showing little to no efficacy over placebo (Traeger et al. 2018), whilst the case series yielded positive, immediate, post-PNE changes in a subgroup of patients (Louw et al. 2019a). On the true preventative side, PNE is now being used and examined in schools within the United States, teaching middle school children about the neurobiology and neurophysiology of pain, with meaningful changes in pain knowledge and healthier beliefs regarding pain (Louw et al. 2018a; Podolak et al. 2019).

In between the various randomised clinical trials, systematic reviews and meta-analyses used to establish the efficacy of PNE, additional clinical studies have emerged to answer various clinical application questions (Louw et al. 2016c, 2017b), for example, the expansion of PNE to conditions other than chronic low back pain, such as chronic whiplash and chronic fatigue syndrome (Meeus et al. 2010; Van Oosterwijck et al. 2011), and the use of telehealth and virtual reality for PNE delivery (Louw 2014; Louw, Louw & Flynn 2019b). Recent research has also focused on identifying which patients would perform well with PNE. Various authors have implied that PNE is best suited for chronic musculoskeletal pain: for patients with high levels of fear-avoidance and/or pain catastrophisation or patients who are ready for change, that is, contemplation and preparation phase of the trans-theoretical model of change (Louw et al. 2017b; Moseley & Butler 2015). One specific indication for PNE is gaining more interest than others – the clinical presence of central sensitisation (CS). It is now well established that a significant part of a person’s pain experience is correlated with the vigilance of the central and peripheral nervous systems (Nijs, Van Houdenhove & Oostendorp 2010; Nijs et al. 2011). Although CS is not directly measurable in humans, various indirect measures are used to suggest CS, including a list of common signs and symptoms and scores in excess of 40 points on the central sensitisation inventory (CSI) (Neblett et al. 2013; Nijs et al. 2010). Central sensitisation is often accompanied by higher levels of fear and pain catastrophisation, higher levels of pain and disability as well as increased healthcare utilisation (Neblett et al. 2013; Nijs et al. 2010).

All of these have been positively influenced by PNE and applied in various conditions known to be associated with CS, including chronic low back pain, fibromyalgia, chronic fatigue syndrome, chronic whiplash associated disorders and more (Louw et al. 2016d; Wood & Hendrick 2019).

With the increased awareness of CS, scientists have now shown that CS is actually quite common in various conditions seen by physiotherapists on a regular basis (Nijs et al. 2010). One such example is shoulder pain. Current studies suggest that a subgroup of patients with shoulder pain present with signs and symptoms consistent with CS (Nijs et al. 2010; Sanchis et al. 2015). With failed conservative care, these patients may end up with shoulder surgery. It has been argued that the presence of CS along with high levels of fear-avoidance and pain catastrophising are associated with poor postoperative outcomes related to persistent pain and disability (Baert et al. 2015; Hirschmann et al. 2013; Theunissen et al. 2012). Pain neuroscience education has been shown to positively influence the sensitivity of the nervous system prior to lumbar surgery and more recently total knee arthroplasty, with improvements in pressure pain threshold (Louw et al. 2015b, 2018b, 2019b).

The aim of this study was to determine whether preoperative PNE would result in any immediate benefit for patients undergoing shoulder surgery.

## Methods

### Patient descriptions and examinations

This case series comprises data from a sample of 12 consecutive patients arriving at an outpatient physical therapy clinic in the United States with shoulder pain and limited range of motion (ROM), awaiting shoulder surgery. Patients meeting the inclusion criteria were sent to a physiotherapist by the surgeon for one preoperative PNE session prior to surgery. Inclusion criteria were that patients had to (1) be scheduled to have shoulder surgery in the next 2 weeks, (2) indicate their willingness to participate in the study and (3) have the ability to read and understand English, as the study included the use of an English educational booklet.

Because all potential participants for our study had been screened and cleared for their orthopaedic surgery, the only exclusion criterion was an unwillingness to participate in the study.

### Self-report outcome measures

Prior to PNE and after completion of the consent and demographic intake forms, patients were asked to complete self-report surveys related to shoulder pain, fear-avoidance, pain catastrophisation, fear of movement and their beliefs about surgical outcome. Patients were also asked to complete the CSI once, prior to PNE, to measure for CS in order to further describe the study population.

- Pain: Self-reported shoulder pain was measured using a Numeric Pain Rating Scale (NPRS), which has been shown to be a valid and reliable measure in patients with shoulder pain (Moseley 2002, 2003, 2005). Whilst the minimal detectable change (MDC) score for patients with shoulder pain has been reported as 2.5 points, the minimal clinical important difference (MCID) for the NPRS has been reported as 1.1 (Mintken, Glynn & Cleland 2009).
- Pain catastrophisation: Pain catastrophisation was measured using the Pain Catastrophising Scale (PCS). The PCS is a self-report questionnaire that assesses inappropriate coping strategies and catastrophic thinking about pain and injury. The PCS has been used in previous pain science studies (Moseley 2004; Moseley, Nicholas & Hodges 2004b) and demonstrated strong construct validity, reliability and stability (Sullivan, Bishop & Pivik 1995). The PCS utilises a 13-item, 5-point Likert scale with higher scores, indicating elevated levels of catastrophising. Previous studies utilising the PCS have shown a median score of 18 in healthy individuals, and a score over 30 was reported as a high level of pain catastrophising (Sullivan et al. 1995). In patients with shoulder pain, the MDC for the PCS is reported to be 9.1 (George, Valencia & Beneciuk 2010) and the MCID has not been established.
- Fear of movement: To evaluate the participant’s pain-related fear of movement and (re)injury, the original 17-item Tampa Scale of Kinaesiophobia (TSK) was used (Cleland, Fritz & Childs 2008; Hapidou et al. 2012). Each item is scored on a 4-point Likert scale that ranges from strongly agree (1) to strongly disagree (4). Total scores range from 17 to 68, and higher scores indicate more fear of movement and/or (re)injury. In patients with shoulder pain, the MDC for the TSK is reported to be 5.6 (Hapidou et al. 2012), and the MCID has not been established.
- Beliefs regarding shoulder surgery: The 12 patients scheduled for shoulder surgery were also asked to rate their level of agreement on a 10-point Likert scale (strongly disagree [0] – strongly agree [10]) with six statements regarding shoulder surgery. The statements were used in a similar PNE study for lumbar surgery and total knee arthroplasty and adapted for shoulder surgery (Louw et al. 2014a; Louw, Diener & Puentedura 2015a).
  I feel prepared and ready to have shoulder surgery.
  I am afraid of the upcoming shoulder surgery.
  I know what to expect after the shoulder surgery.
  Shoulder pain after the surgery is expected.
  I can control the amount of pain I may experience after the surgery.
  The shoulder surgery will fix my pain.
- Central sensitisation inventory: The CSI is a 25-question survey used in screening for CS. The 25-question survey offers answers for each question ranging from 0 to 4 points, with the CSI potential score ranging from 0 to 100 points (Mayer et al. 2012). The CSI has been found to have high reliability and validity, and it is proposed that a score of 40 or above is indicative of CS (Nijs et al. 2010).
- Shoulder flexion active range of motion (AROM): Active shoulder flexion of each patient’s ‘affected’ arm was assessed with a goniometer, with the patient in a seated position. To allow consistency of pre- and post-PNE measurements, skin marks were placed for the goniometric measurements. There is good reliability and validity of goniometric shoulder AROM measurements (Kolber et al. 2012; Salamh & Kolber 2012). The MDC for shoulder flexion has been reported as 8°, and calculation of the MCID is dependent on patient pathology (Kolber et al. 2012).
- Nerve sensitivity: To assess the sensitivity of the nervous system, pressure algometry was used. Pressure pain thresholds (PPTs) followed standardised protocols (Fernandez-de-Las-Penas et al. 2009) and were measured in kilograms per square centimetre (kg/cm^2^) using a digital pressure-pain algometer. The algometer had a 1-cm^2^ round rubber tip that was placed over the three predetermined points of (1) the deltoid insertion of the affected shoulder, (2) the deltoid insertion of the unaffected shoulder and (3) the posterior midline of the dominant-sided knee. Before applying pressure, the examiner instructed each participant: ‘I am going to begin applying pressure to your skin. I want you to tell me the moment the sensation changes from comfortable pressure to slightly unpleasant pain’. Pressure was then applied at a rate of 5 N/s. The examiner stopped applying pressure and recorded the measurement when the participants said ‘now’. Three consecutive PPT measurements were taken at each point with 20 s rest between measurements, and the mean of the three trials was used for analysis. Various studies have reported a 15% reduction in PPT as a significant clinical change (Moss, Sluka & Wright 2007; Sterling, Jull & Wright 2001).

To ensure some level of blinding, all outcome measures, shoulder AROM and PPT were conducted by therapists who did not know what the intervention (PNE) for the study was, and therapists administering the PNE were kept blinded to the pre- and post-PNE measurements. All self-report outcome measures were repeated immediately after the PNE so that they could be compared with pre-PNE scores.

### Intervention: Preoperative pain neuroscience education

The 30-min PNE programme used in this study was an adaptation of the programme developed for lumbar surgery and total knee arthroplasty (Louw et al. 2013, 2014a, 2016a, 2018b, 2019c). The educational material and content used in the previous studies were altered to reflect shoulder pain (Louw 2015). The educational programme was designed to be delivered by a physiotherapist in one-on-one sessions utilising metaphors, examples and images. Patients also received a patient’s booklet containing the same information provided during the one-on-one session. The primary focus of the preoperative PNE session was to help patients re-conceptualise their shoulder pain as an increase in nerve sensitivity and upregulation of the peripheral and central nervous systems, at the same time defocusing attention from the nociceptive input via the tissues from the affected areas. The PNE message thus aimed to reduce anxiety and uncertainty and promote positive expectations and beliefs.

The PNE programme was designed to include prepared pictures (Moseley 2004; Moseley et al. 2004; Van Oosterwijck et al. 2011), examples (Moseley et al. 2004; Van Oosterwijck et al. 2011) and metaphors (Van Oosterwijck et al. 2011). The sensitivity of the nervous system, metaphorically described as an alarm system (Van Oosterwijck et al. 2011), accompanied by drawings of action potentials (Moseley 2004; Van Oosterwijck et al. 2011), was used to describe peripheral sensitisation (Moseley 2004; Van Oosterwijck et al. 2011), CS (Moseley 2004; Moseley et al. 2004; Van Oosterwijck et al. 2011) and plasticity of the nervous system (Moseley et al. 2004; Van Oosterwijck et al. 2011). The PNE sessions for the 30-min research group were delivered by two physiotherapists (D.R. and L.L.) who had completed a 6-month postgraduate pain certification and used PNE on a daily basis in clinical practice.

### Statistical analysis

Data analysis was performed using IBM SPSS Statistics version 25 (SPSS, Chicago, IL, USA). Descriptive statistics were calculated for means and frequencies for the sample population and patient beliefs. A significance level of 0.05 was set for all analyses. Paired-samples *t*-tests were used where all assumptions were met to determine whether there was a statistically significant mean difference between the pre-PNE and post-PNE scores on the TSK, PCS, flexion AROM of the involved shoulder and PPT taken over the involved shoulder, uninvolved shoulder and the knee. Non-parametric tests were performed on the data for pain rating as these data were not normally distributed.

### Ethical consideration

This study was approved by the Internal Review Board (IRB)/Ethics at Southwest Baptist University on 31 January 2017. Patients provided written and verbal consent to participate in the study.

## Results

### Sample description

The mean age of the 12 study participants was 61.7 years (SD = 11.5; range, 42–82 years), and the average number of months that the participants reported being in pain was 26.1 months (SD = 49.6; range, 1.5–180 months). Eight of the study participants (66.7%) were female, and half (50%) of the involved shoulders were on the right side. One hundred per cent of the participants were having shoulder surgery on the involved shoulder for the first time, and three participants (25%) had received shoulder surgery on the other, uninvolved, side previously. The most common pre-surgical diagnosis was rotator cuff tear (*n* = 8 [66.7%]). Additional diagnoses included labral tear, fracture and shoulder osteoarthritis. The mean CSI score for the sample was 33.83 and five patients (41.7%) met or exceeded the score (40 points) indicative of the cut-off for CS.

### Pain rating

The difference in scores for the pre- and post-PNE pain ratings was not normally distributed, as assessed by Shapiro–Wilk’s test (*p* > 0.0005). Therefore, an exact sign test was conducted to determine any effect of the PNE on the NPRS. There was no statistically significant median decrease in the participant’s reported pain rating after receiving the PNE (pre-PNE, 3.5; post-PNE, 3.0; *p* = 0.125). Three patients reported a reduction on the NPRS of 2 points after PNE, exceeding the MCID.

### Tampa Scale of Kinaesiophobia

Participants showed a decrease in the TSK following PNE (33.17 ± 7.14) compared with before PNE (37.17 ± 5.62; range, −14 points to +4 points; median, −4 points), a statistically significant mean decrease of 4.0 points (95% CI, 1.02–6.98; *t* (11) = 2.954; *p* = 0.013; *d* = 0.853; Table 1). The mean post-PNE difference did not meet the MDC of 5.6 points. Four patients (33.3%) experienced a post-PNE TSK reduction in excess of the MDC.

**TABLE 1:** Outcome measures for Tampa Scale of Kinaesiophobia, Pain Catastrophising Scale, shoulder active ROM and PPT.

Outcome measure	Difference of the means	*p*	Effect size Cohen’s *d*
Tampa Scale of Kinaesiophobia	4.00 points	0.013*	0.853
Pain Catastrophising Scale	1.50 points	0.250	NA
Shoulder flexion ROM	5.33°	0.013*	0.856
PPT of involved shoulder	0.89 kg of force	0.004*	1.03
PPT of uninvolved shoulder	0.20 kg of force	0.648	NA
PPT of knee	0.66 kg of force	0.097	NA

NA, not applicable; PPT, pressure pain thresholds; ROM, range of motion.

* , Values that are statistically significant.

### Pain Catastrophising Scale

Participants showed a decrease in the PCS following PNE (7.50 ± 6.52) compared with before PNE (9.00 ± 7.82; range, −12 points to +4 points; median, −1 point), but this difference was not found to be statistically significant. The mean decrease was 1.50 points (95% CI, −1.22 to 4.22; *t* (11) = 1.22; *p* = 0.250), which did not meet or exceed the MDC (Table 1). One patient’s PCS decreased post-PNE beyond MDC.

### Flexion active range of motion

Participants showed an increase in the flexion AROM of the involved shoulder following the PNE (130.4 ± 30.45) compared with before the PNE (135.8 ± 29.89; range, +14° to −6°; median, +5°), a statistically significant mean increase of 5.3° (95% CI, 1.4–9.3; *t* (11) = 2.966; *p* = 0.013; *d* = 0.856; Table 1). The average increase in shoulder flexion AROM did not meet or exceed the MDC for shoulder ROM.

### Pressure pain thresholds

**FIGURE 1:**
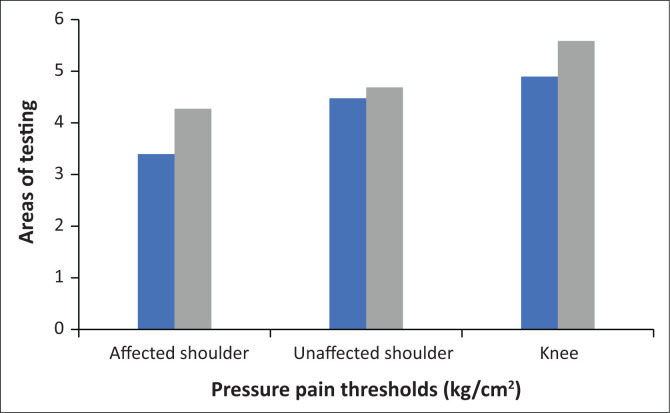
Pressure pain thresholds (kg/cm^2^) before (blue) and after (grey) pain neuroscience education.

Participants showed an increase in the PPT at the involved shoulder following PNE (4.29 ± 2.77) compared with before PNE (3.4 ± 2.34), a statistically significant increase of 0.89 kg of force (95% CI, 0.76–3.18; *t* (11) = 3.574; *p* = 0.004; *d* = 1.03; Table 1; Figure 1). The 26% increase exceeded the MCID for PPT.

Pressure pain threshold at the uninvolved shoulder following PNE increased (4.66 ± 3.52) compared with before PNE (4.46 ± 2.85), but this difference was not found to be statistically significant, and it did not meet the MCID (95% CI, −1.58 to 2.44; *t* (11) = 0.477; *p* = 0.648). Similarly, PPT at the knee increased following PNE (5.59 ± 2.95) compared with before PNE (4.93 ± 2.77), but this difference was not found to be statistically significant and did not meet MCID (95% CI, −0.31 to 3.23; *t* (11) = 1.81; *p* = 0.097).

### Patient beliefs

All the preoperative beliefs regarding surgery shifted positively after PNE, but none reached significance.

## Discussion

To our knowledge, this is the first study exploring the preoperative delivery of PNE for shoulder pain.

Preoperative PNE resulted in no meaningful shifts in pain ratings, fear of movement, pain catastrophisation and AROM; however, a shift was observed through a significant local reduction in nerve sensitivity on the shoulder being operated.

The results from this study concur with the previous preoperative PNE development studies for lumbar surgery and total knee arthroplasty (Louw et al. 2015a, 2015b, 2018b). In all of these studies, this one included, PNE did not result in a significant reduction of pain. In fact, during the development of the PNE for lumbar surgery it was noted that PNE resulted in some slight increases in pain, often referred to as ‘explain pain pain’ (Louw et al. 2015b, 2016b). The inability of PNE to provide a significant immediate change in pain is similar to current education studies, including PNE (Louw et al. 2016d). This may be due to two factors. Firstly, education as a stand-alone treatment has been shown to be not that effective (Gross et al. 2012; Haines et al. 2009). Louw et al. (2016d), in a systematic review of PNE, showed that PNE by itself has little to no effect on pain, whilst PNE combined with other therapeutic treatments, especially movement, yields significant improvements, including reduction in pain. This supports the current PNE+ concept with the ‘plus’ referring to the addition of other treatments along with PNE (Louw et al. 2016d; Marris et al. 2019; Wood & Hendrick 2019). Also, with the introduction of pain as the fifth vital sign and subsequent awareness that repeated enquiry-to-pain ratings and pain-talk (PNE) may in fact increase pain, introduced a phenomenon of ‘explain pain pain’ (Louw et al. 2015b, 2016b).

The current pain neuromatrix theory that describes a distributed neuronal network of processing in various brain areas during a pain experience is likely a key element behind the increased threat appraisal associated with words used by medical providers, including the word ‘pain’ (Louw et al. 2015b, 2016b). In this context and supporting these results, by repeatedly mentioning pain and bringing attention and focus through stories and metaphors, there may have been a heightened awareness and responsiveness to pain.

Our study failed to show any meaningful shifts in scores on the TSK and/or PCS. This concurs with the preoperative lumbar surgery and total knee arthroplasty PNE development studies (Louw et al. 2015a, 2018b). Similar to both previous studies, some patients shift positively, with several patients meeting or exceeding the MDC of the PCS. This may imply that a subgroup that responds favourably to PNE exists; future research needs to investigate if such a group exists and which characteristics constitute that group. It is important to note that mean PCS scores for all the studies were well below the cut-off score for high PCS (30; Sullivan et al. 1995). This is important because high PCS scores have been implicated as a potential indicator of success of PNE (Louw, Nijs & Puentedura 2017a; Louw et al. 2017b). Growing pain science research points to the fact that people with higher scores on the PCS respond more favourably to PNE. Our study’s results would support this notion because some patients did have immediate shifts following PNE. Additionally, there is increased awareness that in surgery a subgroup of patients may be at high risk for poor outcomes when they have high levels of anxiety, depression, fear-avoidance and pain catastrophising (Baert et al. 2015; Hirschmann et al. 2013; Theunissen et al. 2012). It has even been postulated that patients on the higher ends of these spectrums be considered for cognitive behavioural therapy rather than surgery, or at a minimum pre-surgical counselling (Baert et al. 2015; Hirschmann et al. 2013; Theunissen et al. 2012). These results indicate that there’s likely a higher risk subgroup undergoing surgery and PNE (a therapeutic intervention targeting cognitions), which may be more indicated in that population. This discussion similarly applies to fear-avoidance and kinaesiophobia, whereby our study and the knee arthroplasty study showed significant reductions in TSK after PNE, and even though the mean improvement did not reach MDC, there were patients in whom significant improvement was observed. The preoperative lumbar surgery study found the same results using the fear-avoidance beliefs questionnaire, not the TSK.

The biggest positive shift seen in our study is the 26% immediate improvement of PPT on the affected shoulder after PNE. In the knee arthroplasty preoperative PNE study, a similar big shift in PPT was found at the joint that was being operated on (Louw et al. 2018b). This is important because preoperative sensitisation of the nervous system may be a significant contributor to postoperative pain, disability and pain medication use (Yan et al. 2014). This is underscored by the current interest in providing patients pre-emptive (preoperatively) membrane stabilisers (i.e. gabapentin) as a means to calm the nervous system during the perioperative period (Yan et al. 2014; Yu et al. 2013; Zakkar et al. 2013). The emerging body of research showcasing PNE as a means to dampen the sensitivity of the nervous system preoperatively warrants further study, including comparison to medications designed to calm the nervous system, as a potential safer alternative.

Our study has various limitations. There was no control group, which limits our ability to determine if PNE was better than sham treatment or no intervention at all. The CSI was only administered pre-PNE and should have been done post-PNE to determine if PNE could shift patients below the cut-off threshold in lieu of the CS discussions. No long-term follow-up (acute, 6 and 12 months post-operative) was conducted; it should be part of the next phase along with comparing PNE with other or no treatment.

## Conclusion

For this patient cohort, PNE does not appear to significantly influence pain, self-reported pain ratings, fear-avoidance, pain catastrophisation or patient beliefs. A small group of patients was observed to experience clinically meaningful shifts in pain catastrophising and fear-avoidance. Preoperative PNE for shoulder surgery appears to result in significant reduction in nerve sensitivity on the shoulder being operated on.
